# PBJ celebrates twenty years of service to the scientific community by offering free global access, improved ranking and diversity

**DOI:** 10.1111/pbi.13757

**Published:** 2021-12-26

**Authors:** Henry Daniell

**Affiliations:** ^1^ School of Dental medicine University of Pennsylvania Philadelphia PA USA

Welcome to this first issue of the twentieth volume of the *Plant Biotechnology Journal* (PBJ), an open access plant science journal offering free global access to our readers through the open access fee paid by our authors. In this editorial, we recognize our authors, reviewers and editors for their valuable contributions and evaluations in the past nineteen years.

**Figure 1:**
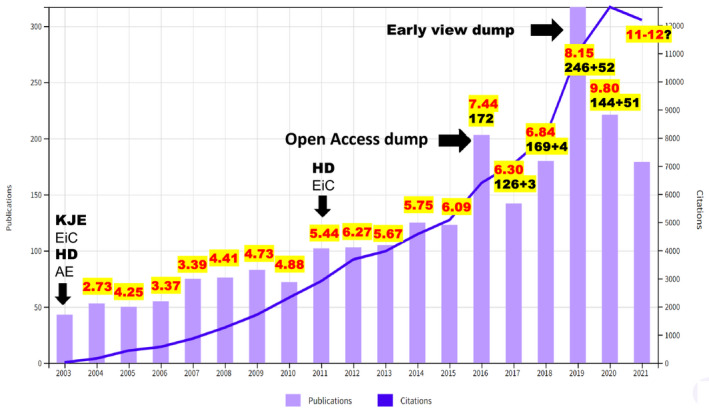
Plant Biotechnology Journal (PBJ) performance history: first issue was published in 2003 with Prof. Keith Edwards (KJE) serving as the first Editor‐in‐Chief and Prof. Henry Daniell (HD) as a founding editor. HD shared the EiC responsibility in 2011 with KJE and assumed full responsibility in 2012. PBJ has grown from publishing 50 articles in 2004 to ~300 articles in 2019, increasing impact factor (IF) from 2.73 to a projected IF >11.0 in 2021. Red numbers show the IF and black numbers show number of articles, including brief communications. Two major events ‘open access dump’ in 2016 by Wiley and ‘Early View dump’ by Web of Science in 2019 increased the number of articles published those years, which impacts IF calculations.

PBJ has grown steadily in the number of articles published from ~50 articles in 2003 to ~300 articles in 2019 (Figure 1). In 2016, PBJ moved away from a subscription print journal to an open access online journal, offering open access for all articles published since inception. Contrary to the anticipated concerns on negative impact or journal growth, PBJ continued to grow by publishing more articles and improving ranking among the plant science or biotechnology journals. PBJ’s impact factor (IF) increased from 2.73 in 2004 to 9.8 in 2020, with anticipated IF of >11 in 2021 (Figure 1). While many high‐ranking journals restrict the number of articles to maintain ranking, PBJ continues to publish more articles, while maintaining a rigorous review process. Irrespective of the evaluation metrics used (IF or CiteScore), PBJ currently ranks third among the plant science journals publishing original research. Scopus CiteScore continues to rank PBJ first among the 334 Agronomy and Crop Science journals.

**Figure 2:**
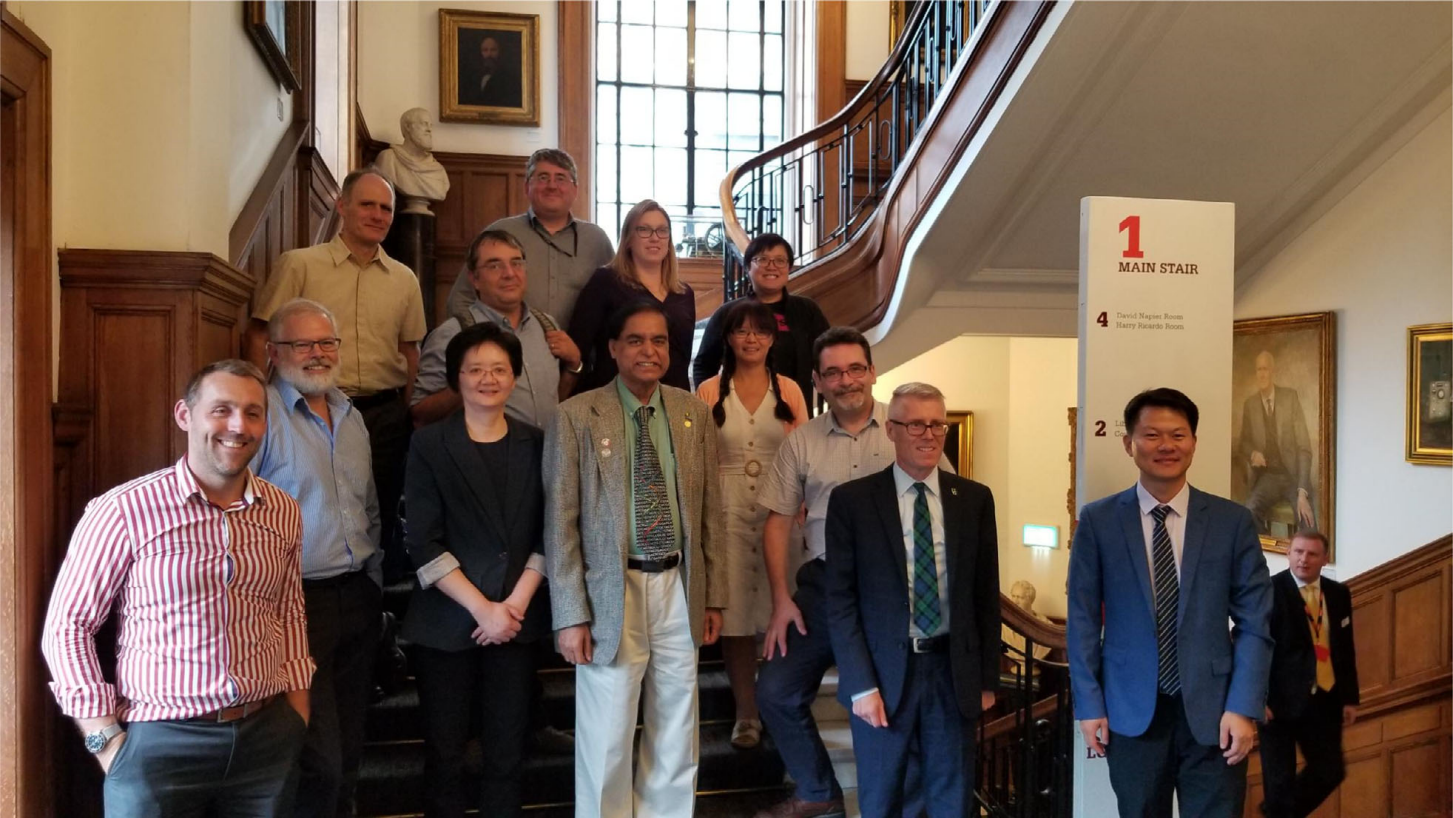
Plant Biotechnology Journal 2019 Editorial Board Meeting in London. Front row: (Adam Wheeler, Wiley), Francois Belzile (AE), Kan Wang (AE), Henry Daniell (Editor‐in‐Chief), Jihong‐Liu Clarke (AE), Dominique Michaud (SE), Malcolm Campbell (AE), Shaungxia Jin (SE); Back row: Stephen Streatfield (AE), Marco Maccaferri (AE), Jim Ruddock (Wiley), Hannah Qualtrough (Wiley) and Caixia Gao (AE).

**Figure 3:**
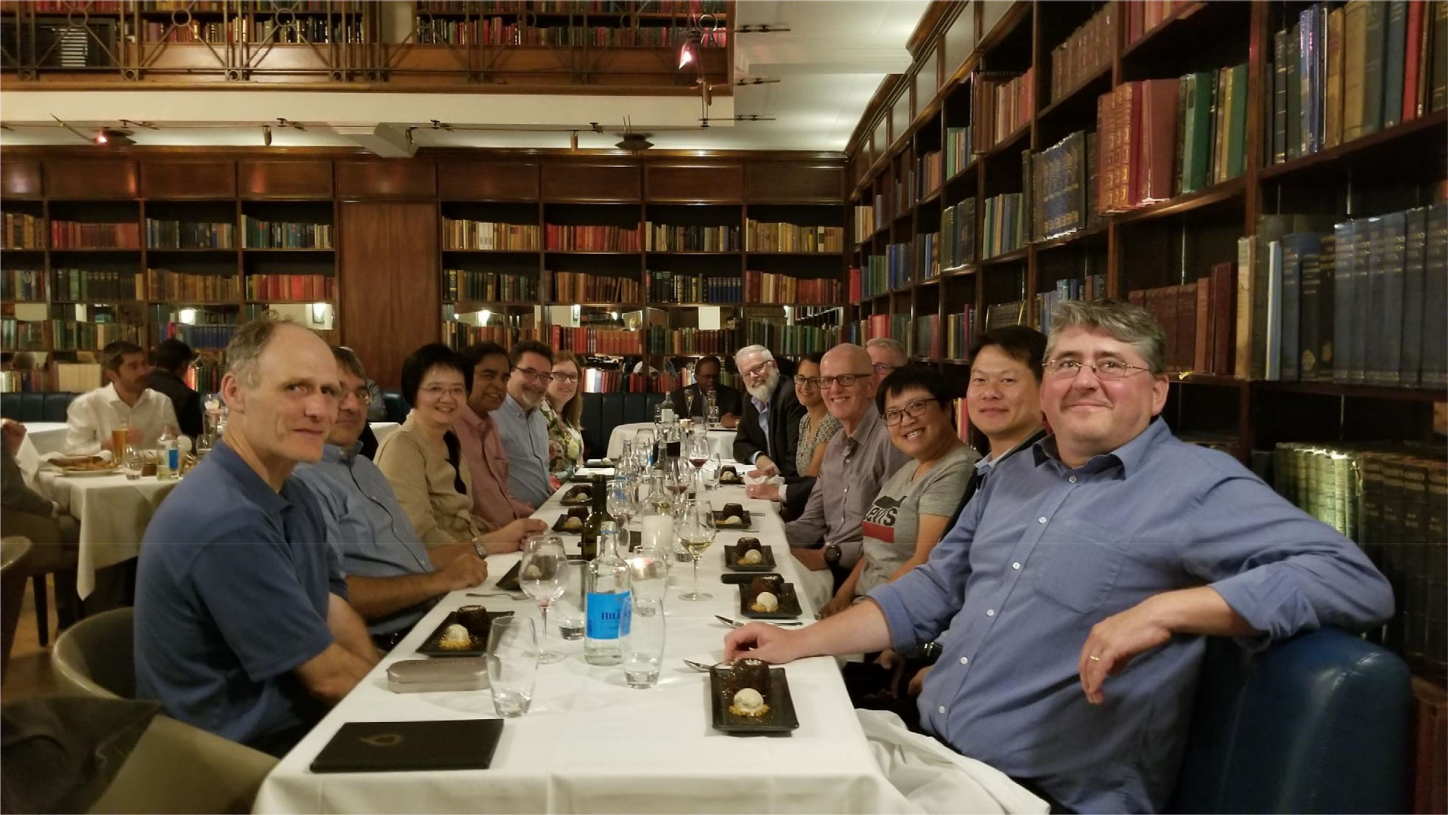
Plant Biotechnology Journal 2019 editorial board dinner in Cinnamon Club, London. Left row: Stephen Streatfield (AE), Marco Maccaferri (AE), Kan Wang (AE), Henry Daniell (Editor‐in‐Chief), Dominique Michaud (SE), Hannah Qualtrough (Wiley). Right row: Jim Ruddock (Wiley), Shaungxia Jin (SE), Caixia Gao (AE), Martin Parry (AE), Malcolm Campbell (AE), Nicola Patron (AE), and Francois Belzile (AE).

**Figure 4:**
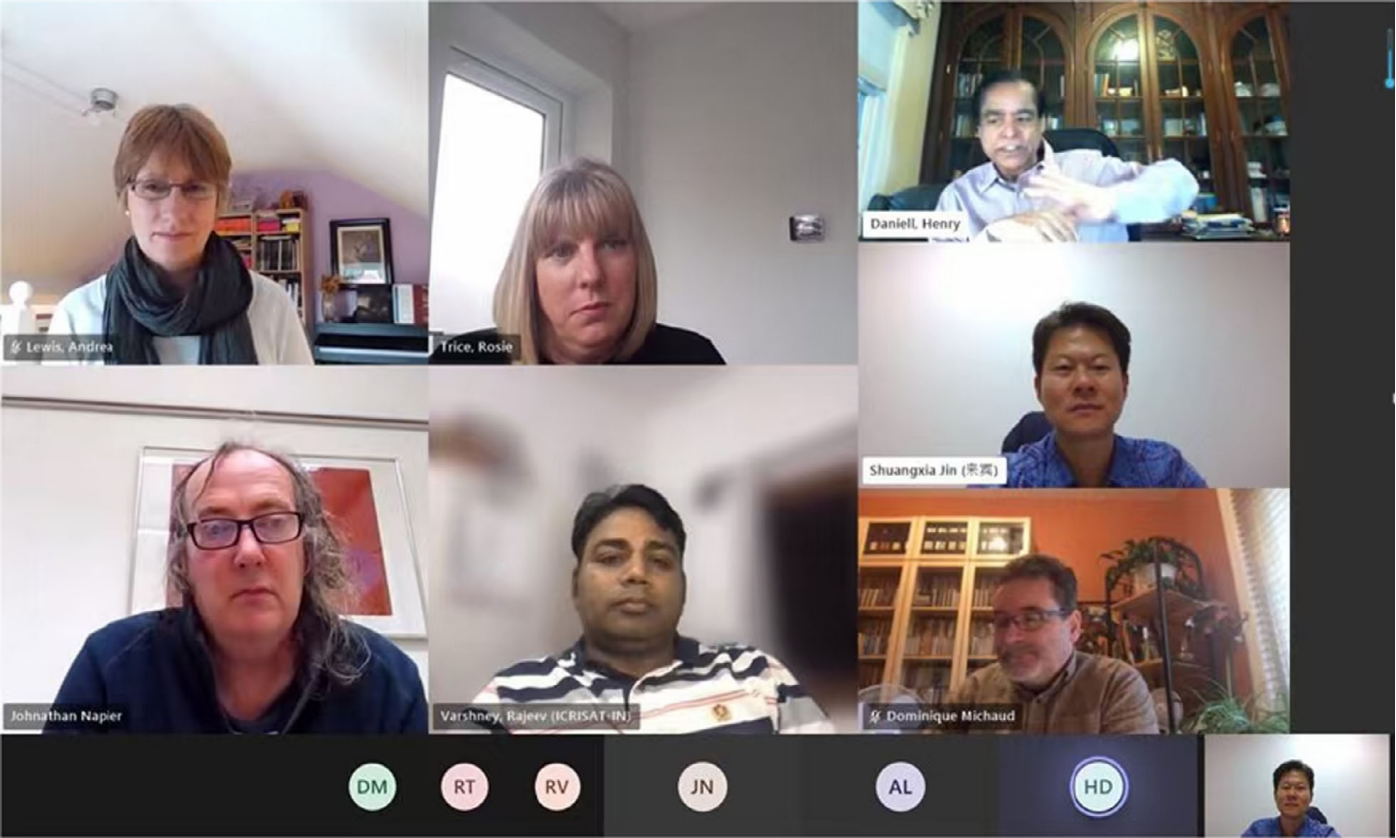
PBJ Editor‐in‐Chief and senior editors meeting with the Wiley management team. Top row: Andrea Lewis (Associate Managing Editor), Rosie Trice (Senior Publishing Manager), Henry Daniell (Editor‐in‐Chief); second row senior editors: Johnathan Napier, Rajjev Varshney, Shuangia Jin and Dominique Michaud.

These accomplishments would not have been possible without the contributions of their best research by our authors and dedicated service of our reviewers and editors. Table 1 shows the list of PBJ editors since inception. Drs. Keith Edwards (2002–2011), Henry Daniell (2002‐present), Robert Birch (2002–2013), Loic Faye (2002–2012), Robert Henry (2002–2014) and Paul Quick (2002–2012) are the founding editors of PBJ. Other associate editors who served PBJ nearly a decade or more include Drs. Dominique Michaud (2007‐present), Malcolm Campbell (2008–2020), Dave Edwards (2011–2021), Neal Stewart (2012‐present) and Stephen Streatfield (2012‐present). I thank all 39 editors (Table 1) for their valuable dedicated selfless service. I welcome new associate editors (Mario Caccamo, Nigel Halford and Wolfram Weckwerth) and senior editors (Shuangxia Jin, Johnathan Napier and Rajeev Varshney), who changed their role this year. Having served PBJ for two decades, I realize that associate editors or senior editors remain anonymous and their services are not adequately recognized. Therefore, brief bios of current associate editors are displayed on PBJ website. Before the pandemic, we were able to meet in London and discuss strategies to advance PBJ (Figures 2, 3). During the pandemic, we meet regularly via zoom (Figure 4). Thanks to Ms. Rosie Trice, Andrea Lewis, Adam Wheeler, Jim Ruddock and Hannah Qualtrough for facilitating these meetings.

**Table 1:** PBJ Associate Editors – past and present

Editor Full Name	Duration of service
An, Gynheung	2007 – 2008
Batley, Jacqueline	2021 – Current
Belzile, François	2018 – Current
**Birch, Robert**	**2002 – 2013**
Caccamo, Mario	2021 – Current
Campbell, Malcolm	2008 – 2021
Chen, Xiao‐ya	2013 – Current
**Daniell, Henry**	**2002 – current**
Davies, Maelor	2010 – 2014
Edwards, David	2011 – 2021
**Edwards, Keith**	**2002 – 2011**
**Faye, Loic**	**2002 – 2012**
Gao, Caixia	2019 – 2020
Halford, Nigel G.	2021 – Current
Hall, Anthony	2020 – 2021
He, Zuhua	2017 – Current
**Henry, Robert**	**2002 – 2014**
Huang, Xuehui	2020 – Current
Jacobs, Thomas B.	2020 – Current
Jin, Shuangxia	2018 – Current
Liu‐Clarke, Jihong	2018 – 2020
Maccaferri, Marco	2018 – 2021
Michaud, Dominique	2007 – Current
Mao, Yanfei	2020 – Current
Napier, Johnathan	2014 – Current
Parry, Martin	2016 – Current
Patron, Nicola	2016 – Current
Petolino, Joseph	2014 – 2016
Qi, Yiping	2020 – Current
**Quick, Paul**	**2002 – 2012**
Stein, Nils	2020 – Current
Stewart, Neal	2012 – Current
Streatfield, Stephen	2012 – Current
Tripathi, Leena	2020 – Current
Varshney, Rajeev	2013 – Current
Wang, Kan	2017 – Current
Weckwerth, Wolfram	2021 – Current
Yang, Bing	2020 – Current
Zhang, Qifa	2013 – 2016
Zhou, Daoxiu	2016 – 2019

Founding editors are indicated by bold letters.

Table 2 shows the list of PBJ reviewers who reviewed more than ten manuscripts in the past ten years. In particular, I thank reviewers who reviewed more than thirty manuscripts (Jacqueline Batley, Henry Daniell, Yiping Li, Wusheng Liu, Xianlong Zhang, Shuangxia Jin and Dominique Michaud), some of whom performed this service in addition to serving as associate editors. Timely reviews amidst the COVID pandemic have significantly decreased the average turnaround time (although few manuscripts were delayed when reviewers/editors experienced COVID‐19), rewarding our authors with timely decisions on their submissions. As the Editor‐in‐Chief, I receive the brunt of criticism from authors for delays in the review process or upon receiving rejection letters and fully understand these challenges. Therefore, offering public service to the scientific community, on top of all other responsibilities, is truly appreciated.

**Table 2:** Plant Biotechnology Journal reviewers (2011–2021)

Name	Completed
**>30 manuscripts reviewed**	
Batley, Jacqueline	39
Daniell, Henry	35
Qi, Yiping	35
Liu, Wusheng	34
Zhang, Xianlong	34
Jin, Shuangxia	32
Michaud, Dominique	32
**>20 manuscripts reviewed**	
Fernie, Alisdair	28
Halford, Nigel G.	28
Puchta, Holger	28
Takaiwa, Fumio	28
Zhang, Baohong	27
Huang, Xuehui	25
Luo, Hong	25
Stoger, Eva	25
Clemente, Tom	24
Mahfouz, Magdy	24
Mason, Hugh	23
Bock, Ralph	22
Henry, Robert	22
Wang, Jiawei	22
Rybicki, Edward	21
Wei, Peng‐Cheng	21
Liu, Ji‐Hong	20
Liu, Qiaoquan	20
**>10 manuscripts reviewed**	
Lee, Keunsub	19
Lenaghan, Scott	19
Mazarei, Mitra	19
Paul, Matthew	19
Raines, Christine	19
Tang, Weihua	19
UNVER, Turgay	19
Yuan, Joshua	19
Zhang, Dabing	19
Zhang, Yong	19
Coleman, Heather	18
He, Zuhua	18
Menassa, Rima	18
Xiao, Han	18
Conrad, Udo	17
Day, Anil	17
Dou, Daolong	17
Guo, HuiShan	17
Kawano, Yoji	17
Xie, Kabin	17
Bansal, Kailash	16
Cheng, Lailiang	16
Lu, Shan	16
Wang, Kan	16
Wang, Kejian	16
Xiong, Lizhong	16
Zhang, Feng	16
Zhou, Huanbin	16
Bai, Songling	15
Bayer, Philipp	15
Haslam, Richard	15
Kumar, Shashi	15
Li, Laigeng	15
Lomonossoff, George	15
Orzaez, Diego	15
Parkin, Isobel	15
Srivastava, Vibha	15
Golicz, Agnieszka	14
Peng, Liangcai	14
Qian, Qian	14
Tuberosa, Roberto	14
Wang, Daowen	14
wang, guodong	14
Zhou, Man	14
Fu, Xiangdong	13
Ma, Zhiying	13
Moriguchi, Takaya	13
Qiu, Jin‐Long	13
Que, Qiudeng	13
South, Paul	13
Wang, Yanpeng	13
Xing, Yongzhong	13
Altpeter, Fredy	12
Christou, Paul	12
D'Aoust, Marc André	12
Foyer, Christine H.	12
Liu, Yule	12
Lozano‐Duran, Rosa	12
Schillberg, Stefan	12
Strauss, Steve	12
Tang, Jihua	12
Tian, Zhixi	12
Bar‐Zvi, Dudy	11
Budak, Hikmet	11
Hood, Elizabeth	11
Kurup, Smita	11
Lakshmanan, Prakash	11
Li, Chengdao	11
Li, Quanzi	11
Li, Zhengguo	11
Liu, Shengyi	11
Liu, Yao‐Guang	11
Lloyd, James	11
Luo, Keming	11
Mascher, Martin	11
McDonald, Karen	11
Ni, Zhongfu	11
Petolino, Joseph	11
Qu, Rongda	11
Wang, Ertao	11
Wang, Nian	11
Wang, Zeng‐Yu	11
Yang, Bing	11
Zhang, Hong	11
Allen, Alexandra	10
Artlip, Tim	10
chong, kang	10
Donini, Marcello	10
Dubos, Christian	10
Edwards, David	10
Eudes, Aymerick	10
Fraser, Paul	10
Fu, Chunxiang	10
Gao, Caixia	10
Gou, Jin‐Ying	10
Harwood, Wendy	10
He, Yuqing	10
Jia, Yulin	10
Kogel, Karl‐Heinz	10
Lin, Choun‐Sea	10
Liu, Junzhong	10
Mori, Masaki	10
Sainsbury, Frank	10
Schiermeyer, Andreas	10
Shi, Huazhong	10
Xia, Guixian	10
Xia, Lanqin	10
Yan, Jianbing	10

Table 3 shows list of top 50 PBJ authors who contributed each more than 500 citations (and published 3 or more papers in PBJ to date). Journals are ranked by citations of published articles, irrespective of how citations are counted (based on all articles by Scopus for 4 years or by selectively excluding certain articles by the Web of Science). Table 3 specifically recognizes authors who consistently contributed their best articles to PBJ in the past two decades and help build this journal. Both editors in chief lead the way by contributing ~2400 citations, followed by more than 1,500 citations contributed by Drs. Barker, Edwards, Henry, Tuberosa and Morell. Several of the top authors have also served PBJ as editors, in addition to submitting their best research. Table 4 shows top 25 institutions of PBJ authors; Chinese Academy of Agricultural Sciences, Chinese Academy of Sciences, Huazhong Agricultural University, and United States Department of Agriculture (USDA) contributed more than 100 articles. CGIAR, University of California System, University of Queensland, CSIRO, CSIC, CAU, CNRS, John Innes Center, USDOE, INRAE contributed >50 articles.

**Table 3:** Highly cited authors who contributed more than 500 citations and published over 3 articles in the Plant Biotechnology Journal (PBJ)

Author name	Number of articles	Times cited to date
**Over 2000 citations**		
Edwards, Keith J.	18	2398
Daniell, Henry	40	2394
**Over 1500 citations**		
Barker, Gary L. A.	12	1916
Edwards, David	33	1616
Henry, Robert J.	21	1603
Tuberosa, Roberto	6	1553
Morell, Matthew K.	9	1503
**Over 1000 citations**		
Salvi, Silvio	5	1457
Huang, Bevan E.	4	1419
Maccaferri, Marco	4	1349
Batley, Jacqueline	22	1275
Varshney, Rajeev K.	33	1242
Zhang, Xianlong	27	1213
Luo, Ming‐Cheng	4	1173
Appels, Rudi	5	1136
Takaiwa, Fumio	18	1130
Whan, Alex	3	1114
Stewart, C. Neal, Jr.	25	1113
Wong, Debbie	3	1112
Christou, Paul	17	1070
Zhang, Hui	9	1052
Zhu, Jian‐Kang	12	1026
**Over 500 citations**		
Coghill, Jane A.	10	997
Fischer, Rainer	16	954
Faye, Loic	11	924
Voytas, Daniel F.	7	874
D'Aoust, Marc‐Andre	12	868
Capell, Teresa	15	868
Waters, Daniel L. E.	7	860
Gomord, Veronique	10	856
Lomonossoff, George P.	13	844
Mao, Yanfei	5	816
Allen, Alexandra M.	8	795
Jin, Shuangxia	20	781
Kim, Ju‐Kon	8	776
Winfield, Mark O.	7	753
Twyman, Richard M.	9	749
Bardor, Muriel	13	732
Langridge, Peter	11	707
Sack, Markus	9	696
Michaud, Dominique	12	682
Sainsbury, Frank	7	674
Stoger, Eva	13	672
Zhang, Heng	3	656
Yang, Bing	10	655
Jung, Harin	7	639
Burridge, Amanda	7	618
Lopato, Sergiy	9	553
Rybicki, Edward P.	11	547
Lerouge, Patrice	9	542
Banakar, Raviraj	3	541
Ha, Sun‐Hwa	4	536

**Table 4:** The Top 25 institutions publishing in the Plant Biotechnology Journal

Institution	Number of articles (2003–2021)
Chinese Academy of Agricultural Sciences	238
Chinese Academy of Sciences	204
Huazhong Agricultural University	139
United States Department of Agriculture (USDA)	101
CGIAR	79
University of California System	73
University of Queensland	69
Commonwealth Scientific Industrial Research Organisation (CSIRO)	68
Nanjing Agricultural University	61
Consejo Superior de Investigaciones Cientificas (CSIC)	58
China Agricultural University	57
Centre National de la Recherche Scientifique (CNRS)	55
John Innes Center	53
United States Department of Energy (DOE)	53
INRAE	52
Rothamsted Research	49
State University System of Florida	48
Zhejiang University	48
Shanghai Institutes for Biological Sciences (CAS)	45
Agriculture Agri Food Canada	41
Institute of Genetics Developmental Biology (CAS)	41
Max Planck Society	39
University of Pennsylvania	39
International Crops Research Institute for the Semi‐Arid Tropics (ICRISAT)	38
Wageningen University Research	38
Cornell University	36

Despite COVID‐19, the number of submissions to PBJ has continued to increase in 2021. PBJ has received manuscripts from over 50 countries in 2021, representing all continents around the globe, highlighting the breadth of countries that submit to PBJ. Readership is also increasing rapidly and readers from over 210 countries have downloaded papers published in PBJ in 2021. We are on track to have over 1.5 million full‐text downloads of PBJ articles in 2021.

**Figure 5:**
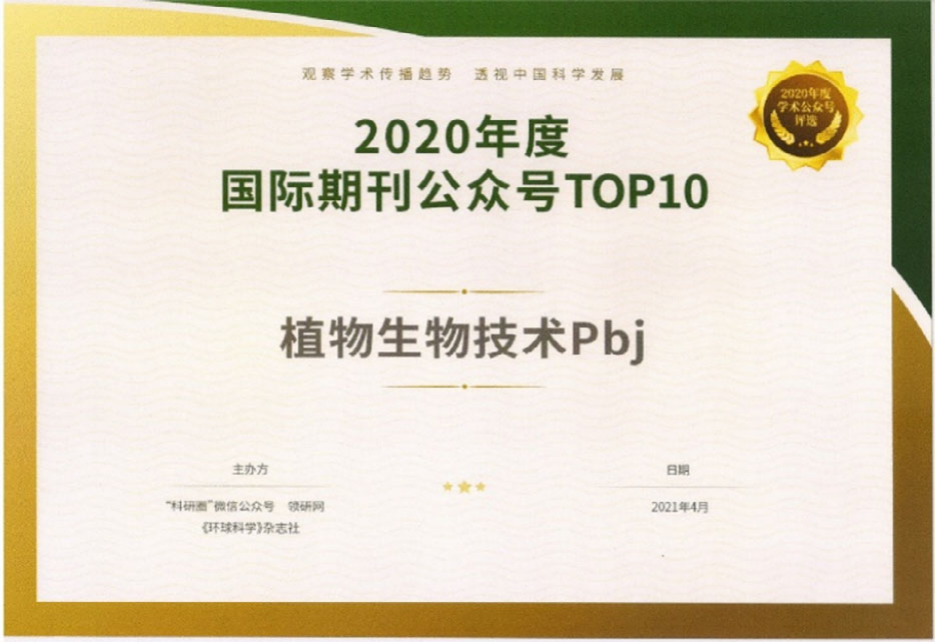
PBJ WeChat account was recognized among the Top 10 academic WeChat accounts of 2020 in China.

PBJ has significantly increased social media activities in 2021, with the help of Professor Shuangxia Jin (PBJ Senior Editor, Huazhong Agricultural University, China) launching the PBJ WeChat account (PBJ ID: PBJ201903) on 1 March 2019. In 2021, this WeChat account has published 2,358 news articles including 371 original articles written by his students and 1987 articles cited from other social media sources. This has resulted in 50,300 followers, with 3,194,774 hits from 1,598,478 computers, 10,200 hits per day and 2,800 hits per news of each publication from *Plant Biotechnology Journal*. In 2021, PBJ WeChat account was recognized among the Top 10 academic accounts of 2020 in China, along with Cell Press, Springer/Nature, Science/AAAS, NEJM, The Lancet, Elsevier, ACS and RSC (Figure 5). In order to enhance communication of complex biotechnology concepts to the public, I am introducing graphical abstracts in 2022 for all full length articles published in PBJ. Likewise, in order to enhance rigor and depth of investigations reported in Brief Communications, I am introducing supplementary data, similar to other high impact plant science journals. In addition, I request that authors provide Twitter messages at the time of manuscript submission. Posts from the PBJ Twitter account @PlantBiotechJ have generated an average daily rate of 120 tweet impressions, for a total of 43 500 impressions over the last 12 months. In addition, I encourage authors to share news releases on their articles with the PBJ editorial office so that they can be included in Wiley Plant Science tweets @wileyplantsci, which currently has more than 16 900 followers.

Ethnicity in scientific journals has emerged as a major concern globally. PBJ has 12 White/13 non‐White Associate Editors when compared to 12 White in Plant Cell, Annual Review of Plant Biology and 13 White/1 non‐White in Plant Physiology. Thus, PBJ has a high diversity of associate editors representing different races, geographical locations and gender balance, although still does not reflect 2019–2020 authorship from American institutions (37 White/62 non‐White corresponding authors and 269 White/403 non‐White co‐authors). PBJ is currently revitalizing the Editorial Board and inviting suggestions for new members to increase diversity further.

**Message** **from the founding Editor‐in‐Chief, Prof. Keith Edwards (2002–2011)**

Dear Henry,

Thank you for the email, I have been watching the PBJs rise with great interest and not without a little pride. Although I along with the then editorial team played its part in launching the PBJ, its recent success has been attributed to the hard work that you and your team have put in; it is this that has made the PBJ the first choice for any plant researcher keen to get their work published in the best, the most prestigious plant journal, so well done. I could see none but you who achieved this amazing feat!

It is interesting to see how applied plant research has changed in the past 20 years. While there is still much to do and discover in terms of basic plant biology, the development of a few core tools such as high‐throughput sequencing/genotyping and CRISPR‐Cas9 has enabled plant scientists working on a host of crop species to deliver varieties with improved agronomic characteristics; for instance, the recently developed wheat lines with reduced levels of asparagine led to significantly lower the levels of acrylamide in cooked foods. Of course, plant scientists, especially applied scientists, do not exist in a vacuum; hence, for me, the most exciting development is the recent suggestion that the UK government intends to facilitate the use of gene editing in agriculture. Although this turn around has been achieved by concerted effort, I personally think that the research published in the PBJ has played a part in producing convincing results. Gene editing is not only safe but is also highly effective in generating plants with properties that are beneficial to human health and wealth. Surely, such developments mean that applied plant research will remain the most exciting biological subject to work in.

**Figure 6:**
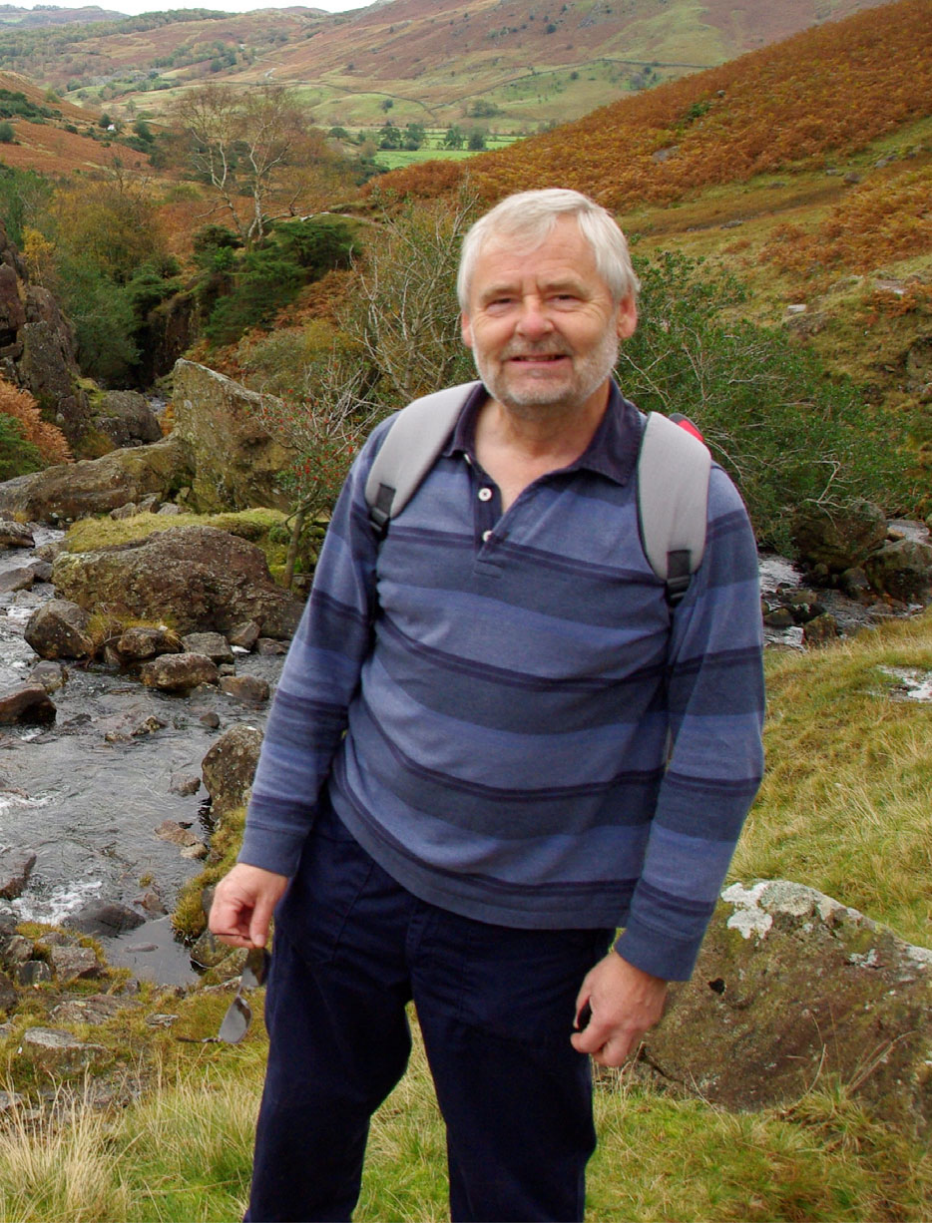
Prof. Keith Edwards, founding Editor‐in‐Chief enjoying his semi‐retirement in the UK’s Lake District.

Hence, do keep up the good work and do let me know what input you might need for your editorial. Recent picture is attached; it is me happily enjoying semi‐retirement in the UK’s Lake District (Figure 6).

Best regards

Keith J. Edwards, Functional Genomics: Office 316, Life Sciences, University of Bristol, 24 Tyndall Avenue, Bristol, BS8 1TQ.

**Message** **from Prof. Martin Parry, Publication Officer, SEB, on launching PBJ**

Dear Henry,

I remember going to Blackwell to pitch the idea of launching the Plant Biotech journal, who were enthusiastic and also keen to involve SEB. Together, we agreed to proceed and were fortunate indeed to have Keith as the first EIC. His careful stewardship ensured the journal made great strides and made my task of convincing the owners to keep faith with the journal much easier. Since you took over, the journal has gone from strength to strength weathering the challenge of going OA without a hitch and PBJ is highly respected within our community. For me, personally it is one of those things that I can be most proud of initiating.

Best,

Prof. Martin Parry, Lancaster Environment Centre, Lancaster University, Lancaster, LA1 4YQ.

Without the outstanding leadership of Ms. Rosie Trice – Senior Publishing Manager at Wiley, Oxford, PBJ would not be able to function and I convey my deepest appreciation, especially on expanding the editorial board. I thank PBJ production editor at Wiley, Ms. Rajalakshmi Sundararamanujam for production from Chennai, India, and timely production and release of PBJ issues every month, Ms. Madhura Amdekar for her expertise in the evaluation of image manipulation, Ms. Reshma Raghu for editorial handling of manuscripts and Ms. Andrea Lewis (Associate Managing Editor) for management. PBJ has also benefitted from the ongoing support from the Society for Experimental Biology and Association of Applied Biologists who co‐own PBJ with the Publisher (Wiley).

PBJ offers several new options for readers to evaluate the short‐ and long‐term impacts of published articles, including Altmetric scores, and Wiley is working towards improving this service by offering details on article readership. I encourage all readers to visit the journal homepage (https://onlinelibrary.wiley.com/journal/14677652) to take advantage of open access, to keep up to date with the latest developments and to sign up for our automated e‐alerts in order to receive emailed notifications when new issues or Early View articles are published. Please note that readers should ‘opt‐in’ to receive e‐alerts, by visiting the journal homepage and registering at the ‘Get Content Alerts’ area.

PBJ management has approved my request to waive or reduce open access fees for manuscripts recommended for publication from authors who do not have adequate funding for publications. Likewise, PBJ waives open access fees for invited reviews and other contributions. I am fully committed to advancing PBJ’s mission of publishing high‐quality manuscripts with free global access, and I look forward to your continued support in 2022.

